# The Impact of COVID-19 on Male Semiprofessional Cricket Player’s Mental Health and Performance Following the Resumption of Sporting Events

**DOI:** 10.1177/15579883231178752

**Published:** 2023-06-07

**Authors:** Lesego S. Malele, Habib Noorbhai

**Affiliations:** 1 Biomedical Engineering and Healthcare Technology (BEAHT) Research Centre, Faculty of Health Sciences, University of Johannesburg, South Africa

**Keywords:** performance, mental health, COVID-19, cricket

## Abstract

The ability of a cricket player to manage their mental health helps them to perform optimally. This study investigated how mental health is related to performance of male cricket players during the resumption of sporting events after coronavirus disease 2019 (COVID-19) restrictions. Mental health profiles were established using the Depression, Anxiety, Stress Scale-21 (DASS-21), Athlete Burnout Questionnaire (ABQ), and Satisfaction with Life Scale (SWLS) instruments among male semiprofessional cricket players (*n* = 63). Performance metrics included: body fat percentage (BF%), range of motion (ROM), push-abdominal test, crazy catch test, *t*-test, 40-m sprint, and Cooper’s test. Inferential statistics included Spearman’s correlations with a significance level set at α < .05. Spearman’s correlation reported a statistically significant relationship for SWLS and body mass index (BMI) (*r* = −0.263; *p* = .037) as well as between stress and abdominal test (*r* = 0.355; *p* = .004); crazy catch test (*r* = 0.249; *p* = .049); Cooper’s test (*r* = 0.335; *p* = .009), VO_2_max (*r* = 0.308; *p* = .014), stress and abdominal test (*r* = −0.313; *p* = .012); as well as anxiety and 40-m sprint (*r* = 0.488; *p* = .027). This study provides an important snapshot of how symptoms of mental health are associated with performance. Further research should investigate the relationship between mental health and performance parameters among male players at varied skill levels.

## Introduction

Mental health is often viewed in isolation rather than as a syndemic factor alongside physical health. As a cricketer progresses in their professional trajectory, a significant amount of time is allocated to being away from their place of residence, which amounts to roughly 300 days per annum (Mahmood & Friedman, 2021). It is evident that athletes in competitive sport are prone to mental health illnesses during times of adversity, such as an injury, stress, social pressure, overtraining, and the requirement for mental toughness despite these pressures (Hundertmark, 2007; Reardon et al., 2019). This can result in depression, anxiety, and eating disorders (Castaldelli-Maia et al., 2019; Souter et al., 2018). All athletes face hardships, challenges, and a range of emotions; therefore, it is important to avoid pathologizing them (Henriksen et al., 2019). A study reported that elite athletes have the same predisposition rate for mental health illness as the general population (Poucher et al., 2021).

Cricket is a sport that encompasses fielding, batting, and bowling, which requires cognitive stimulation (Prakash & Verma, 2022). The sport comprises different formats (i.e., Test, One Day Internationals [ODIs], and Twenty20 [T20]), which has different physiological and psychological demands. As the demands of cricket increase, so does the predisposition to mental health symptoms (Simon et al., 2020). This can be seen through a range of variables, such as sponsorship, training schedules, traveling, and coronavirus disease 2019 (COVID-19). Sports organizations may have systems in place that encourage mental toughness as an ideal, but do not create spaces where elite athletes may open up about their mental health illnesses (Poucher et al., 2021). Formal definitions of life satisfaction differ (Santos, 2013); but life satisfaction involves being content with these aspects of life: emotional, physical, social, and material.

The pandemic of COVID-19, caused by severe acute respiratory syndrome coronavirus 2 (SARS-CoV-2), swept around the globe from Wuhan, China and changed lives (Harding & Noorbhai, 2021; Pillay et al., 2020), economies (Nguse & Wassenaar, 2021) as well as human health (Nassar et al., 2021). In the wake of the pandemic, everyone, including athletes whose lifestyles had been disrupted, has been subjected to soaring mental health stressors (Pillay et al., 2020; Reardon et al., 2019; Uroh & Adewunmi, 2021). To prevent the spread of disease, those who have been exposed to an infectious illness are placed in quarantine, segregated from the general public and forbidden from moving about freely (Magamela et al., 2021). Research on the psychological effects of home confinement owing to a COVID-19 has reported that athletes suffer from these effects (Pillay et al., 2020; Uroh & Adewunmi, 2021).

It has been reported that continuum models cannot tell if the reported symptoms are normal reactions to sports or early signs of mental health difficulties or illnesses (Lundqvist & Andersson, 2021). This was evident in multiple studies: players suffered from mental health illness (Myall et al., 2021); injuries, and a decline in performance due to the tight schedule (Poucher et al., 2021). Prakash and Verma (2022) have posited that data mining techniques play an essential role in ensuring an athlete performs optimally and can detect any anomalies when performance declines. Athletes usually experience demands that are different from the general population, argue Gavrilova and Donohue (2018), and this necessitates treatment that is tailored for their needs.

Mental health profiling is also limited making it necessary for teams to create holistic profiles that are needed to be competitive. Research has identified that COVID-19 increased the phenomenon of mental health disorders, which was evident in an increase in mental fatigue and depression in cricket (Malele & Noorbhai, 2023), football (Bahdur et al., 2022), and other sporting codes (Bansal & Sheriff, 2021; Fröhlich et al., 2021; Grobler et al., 2022). The sedentary behavior that most athletes opted for during lockdown escalated the effects of deconditioning and increase in mental illness (Farren et al., 2018). Studies have reported the challenges college athletes encounter, including inability to resolves their issues (Uroh & Adewunmi, 2021).

The aim of the study was to investigate the relationship between mental health and performance parameters among semiprofessional male cricket players residing in Cape Town, South Africa. Data were collected during the reopening of sporting events after COVID-19 restrictions, between September 2021 and May 2022.

## Methods

### Study Design

This was a cross-sectional research study design. An analytical research method was employed.

### Study Setting and Participants

The research was conducted across five cricket clubs (CCs) from different leagues—Western Province CC (17%), Green Point CC (27%), Milnerton CC (27%), Wynberg CC (21%), and Varsity College CC (8%). Among the selected cricket players (*n* = 63), age ranged from 18 to 35 years with an overall mean and standard deviation of 24.98 ± 5.12 years.

### Study Procedure

The recruitment process included sending coaches and club managers study information via digital and telephonic communication. Informed consents were signed before completing the questionnaires. The participant provided their written consent by signing the consent form provided by the researcher. Upon being granted access, the players were provided with a thorough explanation of the study. The procedure involved the distribution of an information sheet to the participants, who were granted the liberty to terminate their involvement at any given moment. Prior to initiating performance metrics, mental health questionnaires were administered. Three mental health surveys were completed by cricket players via an electronic link and performance metrics was tested by the researcher. Participants were able to go back and change answers if needed on the questionnaire.

### Data Collection

#### Mental Health

More recent studies make use of a 12-item General Health Questionnaire (GHQ-12) (Armino et al., 2021) and Personal Health Questionnaire-9 (Myall et al., 2021). However, influenced by the findings of Vaughan et al.’s (2020) investigation using the Depression, Anxiety, Stress Scale-21 (DASS-21) and Athlete Burnout Questionnaire (ABQ), this study adopted the following mental health instruments: DASS-21; ABQ, and Satisfaction with Life Scale (SWLS). These had been previously validated mental health survey instruments.

##### Depression, Anxiety, Stress Scale-21

The DASS-21: A total of seven items were included in each of the three DASS-21 scales. These subscales were depression, anxiety, and stress. The depression scale evaluates symptoms, such as dysphoria, hopelessness, a low opinion of oneself, a lack of enthusiasm or participation, anhedonia, and laziness. The anxiety scales include measures of autonomic arousal, skeletal muscle effects, situational anxiety, and subjective feelings of anxiousness. The stress scale has an effect on the level of persistent nonspecific arousal. The following are the recommended cut-off scores for traditional severity labels: normal, moderate, and severe (Lovibond & Lovibond, 1995). Prior to interpreting the findings, the values within each subscale were multiplied by a factor of 2, as the DASS-21 represents the abbreviated version of the scale. Subsequently, the evaluations were grounded on the data presented in Table 1.

**Table 1.** Severity of DASS-21 Subscales

Severity	Depression	Anxiety	Stress
Normal	0–9	0–7	0–14
Mild	10–13	8–9	15–18
Moderate	14–20	10–14	19–25
Severe	21–27	15–19	26–33
Extremely severe	28+	20+	34+

##### Athlete Burnout Questionnaire

The ABQ is a 15-item scale that measures the level of athlete exhaustion (Raedeke & Smith, 2001). In the questionnaire for Athletes with Burnout, the condition was characterized by a combination of physical and emotional exhaustion (PEE), a devaluing of sport practice (DSP), and reduced sense of accomplishment (RSA) (Raedeke & Smith, 2001). Cricket players ranked the frequency of their experience on a 5-point Likert-type scale with 1 = almost never, 2 = seldom, 3 = occasionally, 4 = frequently, and 5 = very constantly.

##### Satisfaction With Life Scale

The SWLS assesses the athlete’s cognitive judgment of life, holistically (Diener et al., 1993). The test comprised five questions, which are answered on a scale from 1 to 7. The SWLS Likert-type is an interval scale. Scores from 1 to 1.86 indicate a considerable disagreement. Categorization of the cricketers’ responses followed the Pimentel (2010) study strongly disagree (1.00–1.86), slightly disagree (1.86–2.71), somewhat disagree (2.71–3.57), neither agree nor disagree (3.57–4.43), somewhat agree (4.43–5.29), slightly agree (5.29–6.14), and strongly agree (6.14–7.00).

These three questionnaires have been used in similar studies: DASS-21 (Vaughan et al., 2020), ABQ (Gerber et al., 2018), and SWLS (Litwic-Kaminska & Kotysko, 2022). The survey was designed in a way that to proceed to the next question or section, the cricket player needed to complete the current question. In doing so, it eliminated any blank spaces or risk for nonresponsiveness. The player would have been referred to a psychologist if any extreme anomalies were discovered within the squad.

#### Performance Parameters

Physical testing was conducted from nonfatiguing tests to fatiguing tests (Table 2). These tests are given in Table 2.

**Table 2.** The Sequence of the Fitness Testing Among Semiprofessional Cricket Players

Fitness component	Equipment	Description
Anthropometry: Stature (cm) Body mass (kg) Skinfold (mm)	Seca 213 Stadiometer Seca 813 Robusta Harpenden Skinfold caliper	To assess the cricket player’s height, weight and body fat percentage as part of their demographic profile
Flexibility: Shoulder internal rotation (°) Shoulder external rotation (°) Sit and reach (cm)	SAEHAN goniometer Box and 30 cm ruler	The test is used to measure flexibility of the shoulder internal and external rotation. MSR used to measure flexibility of the lower back and hamstrings
Upper body strength: Seven-stage abdominal test	Mat and 2.5 kg and 5 kg Dumbbell	The ability to complete a sit up with only bodyweight progressing to sit up with a dumbbell
Upper body endurance: 1-min push up	Mat	The ability to complete a sit up with only bodyweight progressing to sit up with a dumbbell
Co-ordination: Crazy catch test	HS Headstart rebounder net	This test is designed to test for hand–eye co-ordination
Agility: Modified *T*-test (MAT) (s)	Cones, stopwatch and 50-m measuring tape	This test is designed to test for change of direction
Speed and acceleration: 40-m sprint (s)	Cones, stopwatch and 50-m measuring tape	The test is used to measure how fast they can cover 40 m
Aerobic capacity: Cooper’s test (min)	Cones, stopwatch and 50-m measuring tape	Measures aerobic fitness of the athlete

### Data Analysis

All participant data were manually entered into Microsoft Excel (2021) using hard copy data sheets from performance-based tests, including morphological measurements and fitness test results. The names of the players were removed and numbering codes were used to refer to the appropriate player. For the mental health screening, the participants’ responses were changed to codes/numbers. The cut-off scores that were specified were utilized to analyze the questionnaires. The cut-off scores were utilized to assign ratings to different symptoms.

The descriptive statistics included differences in positions, age, body fat percentage (BF%), body mass index (BMI), and performance metrics. The performance segment includes descriptive statistics (means ± standard deviations). Inferential statistics included Spearman’s correlations and the Shapiro–Wilk test to suggest the assumption of normality. Reverse coding was used for RSA. The Statistical Package for the Social Sciences (SPSS, IBM Version 27.0) for Windows was used to perform statistical analysis on the data. The level of a significance level was set at α < .05.

### Ethical Considerations

The study was approved by *(anonymized for peer-review; will be added later if the paper is accepted for publication*). Before data collection, all volunteer cricket players provided written consent. In addition, health and safety measures involving sanitization of measurement equipment and the testing site were completed, due to COVID-19 regulations at the time of data collection and testing.

## Results

### Selected Mental Health Parameters

Cronbach’s alpha reported that the three mental health instruments were reliable (Table 3). The DASS-21, ABQ, and SWLS remain among the most dependable instruments for assessing mental health, according to the findings.

**Table 3.** Cronbach’s Alpha Values for the Mental Health Questionnaire

Items	Cronbach’s alpha	*N* of items
Depression, Anxiety, and Stress Scale subscales		
Depression	0.869	7
Anxiety	0.860	7
Stress	0.870	7
Athlete Burnout Questionnaire subscales		
PEE	0.891	5
DSP	0.815	5
RSA	0.833	5
Satisfaction with Life Scale		
SWLS	0.872	5

*Note. N* = number; PEE = physical and emotional exhaustion; DSP = devaluing of sport practice; RSA = reduced sense of accomplishment; SWLS = Satisfaction with Life Scale.

#### Depression, Anxiety, and Stress Scale-21

**Figure 1.**
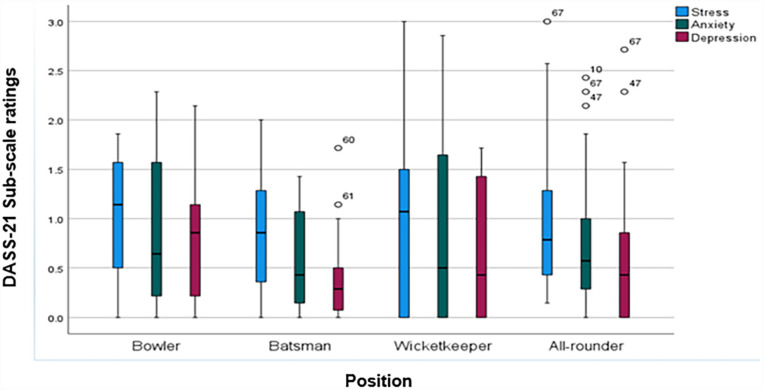
Correlations of DASS-21 Subscales Among Cricketers of Different Positions *Note.* Box indicates the median and interquartile range; whiskers indicate the range. The circles and numbers on the figure represent the outliers (e.g., Player 67).

The following are the recommended cut-off scores for traditional severity labels: normal, moderate, and severe (Lovibond & Lovibond, 1995). Spearman’s correlation indicated that there was a strong, positive, and significant correlation between stress and anxiety (*r* = 0.797; *p* < .000), stress and depression (*r* = 0.763; *p* < .000), and anxiety and depression (*r* = 0.760; *p* < .000). It is also shown in (Figure 1) that (*n* = 3) 4.76% of all-rounders had more outliers compared with other subscales.

#### Athlete Burnout Questionnaire

Spearman’s correlations indicated that there was a moderate, positive, and significant correlation between PEE and DSP (*r* = 0.474; *p* < .000), PEE and RSA (*r* = 0.461; *p* < .000), and DSP and RSA (*r* = 0.496; *p* < .000).

#### Satisfaction With Life Scale

Spearman’s correlation indicated that there was a moderate, negative, and significant correlation between SWLS and stress (*r* = −0.457; *p* < .001); SWLS and anxiety (*r* = −0.373; *p* = .003). However, there was a strong, negative, and significant correlation between SWLS and depression (*r* = −0.506; *p* < .001).

### Selected Performance Parameters

A parametric correlation investigated the relationship between VO_2_max and abdominal test. The relationship was positive, weak in strength, and statistically significant (*r* = 0.275; *p* = .033). This indirectly suggests that VO_2_max is associated with abdominal strength. The results of the other performance metrics were not statistically significant.

### Selected Mental Health and Performance Parameters

#### Depression, Anxiety, and Stress Scale-21

Spearman’s correlation investigated the relationship between stress, anxiety, and depression to physical metrics. There was a weak, negative, and statistically significant correlation between stress and abdominal test (*r* = −0.313; *p* = .012), Cooper’s test (*r* = −0.392; *p* = .002), and VO_2_max (*r* = −0.348; *p* = .005). There was a positive and statistically significant correlation between anxiety and 40-m sprint (*r* = 0.488; *p* = .027) (Table 4).

**Table 4.** Correlation of Mental Health Screening Items With Performance Metrics

Items	Performance metrics							
	Sit and reach	Abdominal test	Push up	Crazy catch test	MAT	40-m sprint	Cooper’s test	VO_2_max
Correlations between DASS-21 and performance metrics								
Stress	0.02	−0.313*	0.09	−0.10	−0.02	0.19	−0.392**	−0.348**
Anxiety	0.12	−0.18	−0.05	−0.16	0.03	0.288*	−0.25	−0.20
Depression	−0.01	−0.285*	−0.01	−0.06	0.03	0.16	−0.439**	−0.381**
Correlations of Athlete Burnout Questionnaire (ABQ) subscales with physical performance								
PEE	0.021	−0.364**	0.029	−0,151	0.090	0.187	−0.207	−0.207
DSP	0.024	−0.366**	−0,111	0.020	0.182	0.037	−0.326*	−0.326*
RSA	0.034	−0.264*	−0.064	−0.208	0.092	0.294*	−0.334**	−0.334**
Correlations of Satisfaction with Life Scale (SWLS) with physical performance								
SWLS	0.08	0.355**	−0.03	0.249*	−0.01	−0.16	0.335**	0.308*

*Note.* Finally, there was a weak, negative and statistically significant correlation between depression and abdominal test (*r* = −0.285; *p* = .023); Cooper’s test (*r* = −0.439; *p* < .001) and VO_2_max (*r* = −0.381; *p* = .002). PEE = physical and emotional exhaustion; DSP = devaluation of sports practice; RSA = reduced sense of accomplishment; SWLS = Satisfaction with Life Scale; MAT = modified agility test.

* Correlation is significant at the 0.05 level (two-tailed). **Correlation is significant at the 0.01 level (two-tailed).

#### Athlete Burnout Questionnaire

There was an inverse significant relationship between the PEE and abdominal test (*r* = −0.364; *p* = .003). There was an inverse significant relationship between the DSP and abdominal test (*r* = −0.366; *p* = .003), Cooper’s test, and VO_2_max (*r* = −0.326; *p* = .011) (Table 4).

There was an inverse significant relationship between the RSA and abdominal test (*r* = −0.264; *p* = .036), Cooper’s test, and VO_2_max (*r* = −0.334; *p* = .009). The study reported a positive significant relationship between RSA and 40-m sprint (*r* = 0.294; *p* = .024).

#### Satisfaction With Life Scale

Spearman’s correlation reported that there was weak positive correlation meaning that the cricket players’ satisfaction is associated with their performance (Table 4). Spearman’s correlation reported a negative, weak in strength, and statistically significant relationship for SWLS and BMI (*r* = −0.263; *p* = .037). There was a weak, positive, and statistically significant correlation between stress and abdominal test (*r* = 0.355; *p* = .004); crazy catch test (*r* = 0.249; *p* = .049), Cooper’s test (*r* = 0.335; *p* = .009), and VO_2_max (*r* = 0.308; *p* = .014).

## Discussion

The aim of the present study was to investigate the mental health profiles and the association of the cricketer’s performance in the light of the pandemic. The study explored selected mental health, selected performance parameters, as well as the relationship between mental health profiles and performance metrics. As a result of orthopedic injuries, a subset of the participants (*n* = 5; 8%) were unable to complete certain tests. The study reported a Cronbach alpha value of ≥ 0.80 which means the questionnaires were reliable. The current study is supported by Walton et al. (2020) based on the Cronbach alpha greater than 80. According to the DASS-21, there was a substantial, positive correlation between subscales and cricket players with high anxiety levels reporting high levels in performance measures. A relation of satisfaction with life and performance metrics was reported by the study.

### Selected Mental Health Parameters

#### Depression, Anxiety and Stress Scale-21

There are limited studies that have investigated mental health variability between elite, amateur, and nonathletes (Vaughan et al., 2020). According to Vaughan et al. (2020), women outperformed men on the DASS-21. The current study reported that the symptoms of depression, anxiety and stress are interrelated. While injured athletes outperformed nonathletes, those with more expertise scored higher on the general factor and depression, and those with less expertise and knowledge scored higher on anxiety and stress (Vaughan et al., 2020). This is in line with the current study as symptoms of anxiety were moderate and symptoms of stress had a high normal based on the DASS-21 cut-off score. Magamela et al. (2021) reported that many people had to live alone and away from their families to stop the spread of the epidemic. The effects of COVID-19 restrictions might have been the catalyst to progressive decline in performance, heightened anxiety as well as changes in body composition.

There was a difference when DASS-21 was compared between recreational athletes and elite athletes (Tubić et al., 2022). Results in the study support the use of DASS-21 in a sport context, therefore providing researchers with a reliable and valid mental health testing instrument. Sportsmen can be can aided with skills to cope with the unique problems they confront if there is an understanding of personality features that may shield them from anxiety and depression (Myall et al., 2021). The current study reported DASS-21 to be based on position rather than personality of people. However, due to a lack of baseline data, it is difficult to compare these findings with prepandemic mental health profiling (Simon et al., 2020). It is common to experience mental and physical depletion as well as a loss of interest and pleasure in one’s work when depressed or burnt out (Raedeke, 1997).

According to Vaughan et al. (2020), depression subscale has received little attention compared with the other two DASS-21 subscales (stress and anxiety). The DASS-21 is yet to be used in a sport context, with the gap first addressed by Vaughan et al. (2020).

#### Athlete Burnout Questionnaire

Depression, which is frequently linked to burnout in athletes, could have a negative impact on performance (Grobler et al., 2022). The current study reported on the interrelationship between ABQ and DASS-21: heightened symptoms of DASS-21 can influence symptoms of ABQ, which could impair performance. Studies have reported that what nonathletes or recreational athletes perceive as challenging, is perceived as milder to elite athletes (Tubić et al., 2022). Hence, the results of this study must be viewed with caution.

#### Satisfaction With Life Scale

Athletes discovered that not being able to participate in sports had a significant negative impact on their level of life satisfaction (Kuok et al., 2021), this study is in line with the current study as cricket players neither agreed nor disagreed they were satisfied with life. More research is needed to understand reasons for dissatisfaction so that tailored interventions are implemented (Schuring et al., 2017). This study might provide insights on how a player’s satisfaction could be associated with their performance.

### Selected Performance Parameters

No significant differences were reported among cricketers between positions for speed (Weldon et al., 2021). It is reported that batters had a higher aerobic base due to running between wickets at a higher intensity (Weldon et al., 2021). The study reported abdominal test and VO_2_max were positive, weak in strength and statistically significant. When compared with the longer format, the T20 had higher intensity and required higher aerobic capacity (Petersen et al., 2009).

### Selected Mental Health and Performance Parameters

#### Depression, Anxiety, and Stress Scale-21

Longer formats of the game are reported to be more physically demanding (Weldon et al., 2021). The current study observed that when stress symptoms worsen, aerobic capacity may suffer. This might be attributable to the effects of COVID-19 sedentary behavior among the semiprofessional cricketers (Farren et al., 2018). Training time decreased from 3.1 (±1.4) h per day before the lockdown to 2.5 (±1.2) h per day, which was statistically significant (*p* = .03) (Fröhlich et al., 2021). As the rules of lockdown were eased, the tight playing schedules coupled with bio-bubbles increased the incidence rate of injuries, mental health illness, and decrease in performance (Pillay et al., 2020).

The current study reported symptoms of anxiety to optimize 40-m sprinting. Symptoms of anxiety a player experiences before or during a sporting event are different from “normal anxiety” (Vaughan et al., 2020). Having enough stimulation is paramount for sprinting abilities. Younger adults were discovered to be more susceptible to anxiety and depression according to Myall et al. (2021), which aligns with this study, which reported that moderate symptoms of anxiety were associated with improvement in sprinting ability. The average age for the study was 19 years. Amateur athletes are more susceptible to anxiety because of their lack of experience in competitions and in controlling arousal (Singh, 2021). However, this investigation reported anxiety symptoms to be a performance-enhancer, rather than hampering performance.

When comparing elite athletes and recreational athletes, the perceived intensity of stressors varies, with intense stressors being mild for elite athletes (Tubić et al., 2022). Getting the right amount of arousal is paramount for the athlete to perform well in the semiprofessional/amateur context. This study reported symptoms of depression affected abdominal strength and aerobic capacity. However, the absence of opportunities to succeed can lead to psychological distress for an athlete or any other person (Kuok et al., 2021). This is vital in the context of the growing popularity of T20, the shortest and fastest form of cricket, due to it being the most lucrative for players, administrators, coaches, and owners, as well as a popular format for attracting fans (Noorbhai & Noakes, 2018; Petersen et al., 2009; Prakash & Verma, 2022).

#### Athlete Burnout Questionnaire

Moen et al. (2019) reported that there are limited studies investigating how psychological aspects lead to athletic burnout. Gümüşdağ (2013) reported that competitive anxiety was significantly associated with burnout. Constant monitoring by a sports psychologist of the mental health of athletes in the team will improve performance. Symptoms of depression were the strongest predictor of a diminished sense of accomplishment and sport devaluation subscales, casting doubt on the convergent validity of the scales (Vaughan et al., 2020). Despite this, research indicates that athlete burnout and mental health are closely linked.

According to Gümüşdağ (2013), burnout was associated with age and competitive anxiety. The study reported that there is a relationship between symptoms of RSA with reduction of aerobic capacity and abdominal test. Symptoms of RSA were associated with improvement in sprinting ability. The onset of COVID-19 increased the demand for play as playing schedules were compressed, potentially leading to an increase in physical and emotional weariness (Bahdur et al., 2022). Despite this, “hardware” (physiology and biomechanics) is the main focus of sports, while “software” (mental health and performance psychology) is often overlooked.

It has been reported that if an athlete is a perfectionist in training it is a trigger for burnout; however, the risk of burnout is doubled if the coach/Biokineticist is also a perfectionist when executing tasks (Olsson et al., 2022). According to Schuring et al. (2017), mental health problems affect as many as 38% of current South African male and female cricketers. According to the researcher, this is the first study to investigate athlete burnout among semiprofessional cricketers. Mental health profiling among cricketers is unknown (McCabe et al., 2021; Ogden et al., 2022; Poucher et al., 2021; Reardon et al., 2019). In the athletic population, burnout is generally perceived as a physical component; however, assessing the psychological aspect will provide causes and potential treatments.

#### Satisfaction With Life Scale

Previous cricket studies have either focused on one position or pooled positional data, making it difficult to report separately reported positional body composition data (Weldon et al., 2021). The current study reported on the association between SWLS and improvement in BMI. This provides snapshots of how body composition of a cricketer improves life satisfaction. Batters have been the subject of sparse scientific study, but it has been hypothesized that improving their upper-body strength, grip strength, rotational power, balance, and proprioception can boost their batting performance (Weldon et al., 2021). This might suggest that an increase in symptoms of SWLS might result in an increase in co-ordination (crazy catch), aerobic capacity, as well as abdominal strength.

According to reports, the intensity, frequency, and level of competition has an effect on the athlete’s quality of life (Santos, 2013); and has been neglected in African literature. Uroh and Adewunmi (2021) reported that elite athletes’ mental health is understudied in comparison with the general population due to the belief that athletes must be tough. Uroh and Adewunmi (2021) established that there are limited studies in Africa exploring how mental health affects performance during the pandemic.

### Limitations

Competitive season shifts necessitated games having to be played quickly. As a result, correlation analysis could only be performed on cricket players who had already completed the mental health questionnaire. Mental health and fitness tests could not be analyzed for the whole season, from preseason to competitive season due to the effects of the pandemic.

### Future Research Directions

- Investigate how anxiety regulation can enhance cricket players’ performance.
- Further investigate the relationship between mental health and performance parameters among cricket players at varied skill levels.
- Examine the lengthier formats of cricket and compare mental health profiles with position-based performances.

### Recommendations

According to the findings of this investigation, it is imperative to:

- Monitor mental health variability weekly.
- Establish a medical team that includes a Mental Health Consultant for cricket players.
- Develop, validate and implement a mental health framework as well as normative data for cricket players.

### Conclusion

This study provides a snapshot of how symptoms of mental health are associated with performance. The creation of a new role for a Mental Health Consultant in cricket (or any sport) teams would be pivotal in ensuring that mental health receives the same attention as the physical aspect of sport, such as performance optimization and/or injury prevention. With the growing popularity of cricket in North America, as well as with the imminent Major League Cricket (MLC) T20 competition, this paper serves as a benchmark for further mental health research among male athletes.
